# Supplementary material for: MetaRibo-Seq measures translation in microbiomes
Source: Nat Commun. 2020 Jun 29;11:3268. doi: 10.1038/s41467-020-17081-z (PMC7324362; doi:10.1038/s41467-020-17081-z)
Supplement: Supplementary file 10 — Supplementary Data 7 [file 41467_2020_17081_MOESM10_ESM.zip › File2/Confidence_High_Taxonomy/132229_out.krona.html]

Javascript must be enabled to view this page.

members
magnitude
magnitudeUnassigned
count
unassigned
taxon
rank

132229\_out

102

superkingdom
102
2

976
1
phylum

1
class
200643

1
order
171549

1
family
815

genus
1
816

818

SRS052027\_contig\_number\_9451
1
species

1239
phylum
101

186801
class
101


SRS011134\_contig\_number\_28740SRS013687\_contig\_number\_contig-100\_26430.117330SRS013940\_contig\_number\_contig-100\_31493.31494SRS015190\_contig\_number\_contig-100\_3783.3783SRS015217\_contig\_number\_633SRS015578\_contig\_number\_contig-100\_11453.11453SRS018817\_contig\_number\_contig-100\_29426.64666SRS046712\_contig\_number\_contig-100\_7596.7597SRS050998\_contig\_number\_2393SRS053398\_contig\_number\_contig-100\_8111.8111SRS063985\_contig\_number\_contig-100\_41557.81377SRS074964\_contig\_number\_contig-100\_35184.35184SRS075984\_contig\_number\_contig-100\_21876.61411SRS097920\_contig\_number\_contig-100\_44608.82971SRS1041031\_contig\_number\_19528SRS1055056\_contig\_number\_contig-100\_10681.10682SRS140492\_contig\_number\_contig-100\_27266.110709SRS144506\_contig\_number\_13416
186802
101
order
18

family
65
541000

genus
65
8
1263

SRS016753\_contig\_number\_contig-100\_8815.8815SRS018623\_contig\_number\_contig-100\_17203.64726SRS019030\_contig\_number\_contig-100\_11624.11624SRS043411\_contig\_number\_9584SRS056273\_contig\_number\_27593SRS063040\_contig\_number\_50241SRS075716\_contig\_number\_contig-100\_15791.15792SRS076976\_contig\_number\_3085


SRS077454\_contig\_number\_16368
2293151
1
species

species
3
2293232

SRS017521\_contig\_number\_45949SRS049402\_contig\_number\_contig-100\_3333.95355SRS105153\_contig\_number\_31684

2293158

SRS051610\_contig\_number\_contig-100\_30127.30128SRS100021\_contig\_number\_contig-100\_1732.41759SRS142923\_contig\_number\_contig-100\_8896.8897
species
3

2293183

SRS013158\_contig\_number\_contig-100\_836.836SRS023715\_contig\_number\_contig-100\_27541.68402SRS148721\_contig\_number\_35701
species
3

2
species
2293208

SRS144362\_contig\_number\_37837SRS893259\_contig\_number\_4809


SRS020233\_contig\_number\_14503
1897020
species
1


SRS149879\_contig\_number\_27410
2302958
species
1

2
species
2293192

SRS018836\_contig\_number\_488SRS104311\_contig\_number\_45958

2293198

SRS049900\_contig\_number\_2080SRS077502\_contig\_number\_13145
species
2

species
1

SRS046717\_contig\_number\_contig-100\_10730.55420
2293244

2293212

SRS1041033\_contig\_number\_8945
1
species

2293241

SRS011302\_contig\_number\_7984SRS017916\_contig\_number\_17947SRS018984\_contig\_number\_16496SRS023526\_contig\_number\_9550SRS043701\_contig\_number\_1345SRS053214\_contig\_number\_18004SRS1041090\_contig\_number\_contig-100\_1955.34305SRS143342\_contig\_number\_15658SRS148159\_contig\_number\_57583
species
9

2293176

SRS015431\_contig\_number\_35706SRS018351\_contig\_number\_16813SRS019068\_contig\_number\_contig-100\_422.655793SRS023914\_contig\_number\_contig-100\_1003.144713SRS042284\_contig\_number\_contig-100\_1792.43703SRS098571\_contig\_number\_7088
species
6


SRS017433\_contig\_number\_1747SRS024075\_contig\_number\_contig-100\_4217.82738SRS104036\_contig\_number\_6545SRS1041091\_contig\_number\_20681SRS1041138\_contig\_number\_7913SRS104485\_contig\_number\_5198SRS104912\_contig\_number\_19959SRS971275\_contig\_number\_23962
2293224
8
species

2293215

SRS014923\_contig\_number\_38171SRS015854\_contig\_number\_15253SRS064276\_contig\_number\_9030SRS076929\_contig\_number\_12548SRS077552\_contig\_number\_24399SRS098514\_contig\_number\_contig-100\_564.564SRS098644\_contig\_number\_10662SRS098717\_contig\_number\_contig-100\_30214.82939SRS104636\_contig\_number\_contig-100\_32863.81627SRS143991\_contig\_number\_24818SRS144297\_contig\_number\_contig-100\_2161.79312SRS148253\_contig\_number\_8795SRS893252\_contig\_number\_15553
species
13


SRS049712\_contig\_number\_contig-100\_189.134768
2293225
1
species

18
family
186803

18
genus
572511

1805476

SRS015782\_contig\_number\_9256SRS150029\_contig\_number\_17819
species
2


SRS017191\_contig\_number\_10517SRS050752\_contig\_number\_contig-100\_555.173933SRS050941\_contig\_number\_19431SRS077127\_contig\_number\_contig-100\_14010.14011SRS078419\_contig\_number\_13922SRS098061\_contig\_number\_contig-100\_20317.58549SRS142503\_contig\_number\_52369SRS142712\_contig\_number\_contig-100\_13138.13138SRS144183\_contig\_number\_23328SRS146812\_contig\_number\_contig-100\_15273.162054SRS148511\_contig\_number\_16917SRS893270\_contig\_number\_15461SRS893378\_contig\_number\_contig-100\_1172.43489
40520
13
species

species
3

SRS016495\_contig\_number\_4580SRS054590\_contig\_number\_contig-100\_5875.5875SRS147139\_contig\_number\_29350
418240
